# 3'-Ethynylcytidine, an RNA polymerase inhibitor, combined with cisplatin exhibits a potent synergistic growth-inhibitory effect via Vaults dysfunction

**DOI:** 10.1186/1471-2407-14-562

**Published:** 2014-08-04

**Authors:** Hiroto Fukushima, Tetsuya Abe, Kazuki Sakamoto, Hiroaki Tsujimoto, Shinji Mizuarai, Shinji Oie

**Affiliations:** Biomarker Research, Tsukuba Research Center, Taiho Pharmaceutical Co., Ltd, 3 Okubo, Tsukuba, Ibaraki, 300-2611 Japan

**Keywords:** ECyd, Vaults, Cisplatin, Biomarker, Resistance

## Abstract

**Background:**

We previously reported that 3'-ethynylcytidine (ECyd, TAS-106), an RNA polymerases inhibitor, enhances the anti-tumor efficacy of platinum in several tumor types in both *in vitro* and *in vivo* tumor models. However, the molecular mechanisms underlying the ECyd-induced enhancement remain elusive.

**Methods:**

Cisplatin (CDDP)-resistant head and neck cancer KB cells were established by stepwise dose escalation with CDDP. The combination effect of ECyd and CDDP were assessed using isobologram analysis. The transcriptional and post-translational statuses of several molecules were detected using real-time PCR, immunoblot analysis and immunocytochemistry. Xenograft assays were used to confirm the mechanisms underlying the ECyd induced enhancement of CDDP anti-tumor efficacy *in vivo*.

**Results:**

ECyd sensitized KB to CDDP by inhibiting the drug transporter Vault complex (Vaults). First, we showed that Vaults were overexpressed in CDDP-resistant KB cells. The suppression of major vault protein (MVP) by RNA interference restored the sensitivity to CDDP. Next, we showed that ECyd significantly sensitized the resistant cells to CDDP, compared with the parental paired cell line. A molecular analysis revealed that ECyd inhibited the synthesis of vRNAs as well as the induction of MVP, both of which are critical components of Vaults as a drug transporter. Furthermore, we found that the synergistic effect of ECyd and CDDP was correlated with the MVP expression level when the effect was analyzed in additional cancer cell lines. Finally, we demonstrated that ECyd decreased the vRNAs expression level in xenograft tumor.

**Conclusions:**

Our data indicated the ability of ECyd to cancel the resistance of cancer cells to CDDP by inhibiting the Vaults function and the decrease of Vaults expression itself, and the ability of the combination therapy with CDDP and ECyd to offer a new strategy for overcoming platinum resistance. Moreover, the study results suggest that Vaults could be a biomarker for stratifying patients who may benefit from the combination therapy with ECyd and platinum.

**Electronic supplementary material:**

The online version of this article (doi:10.1186/1471-2407-14-562) contains supplementary material, which is available to authorized users.

## Background

1-(3-*C*-Ethynyl-s-D-*ribo*-pentofuranosyl)cytosine (3'-ethynylcytidine, ECyd, TAS-106) (Additional file 1: Figure S1A) is an antitumor cytidine analogue possessing potent cytotoxic and antitumor activities in preclinical therapeutic models via the inhibition of RNA biosynthesis through the competitive inhibition of RNA polymerase I, II and III. When administered, ECyd is initially phosphorylated by uridine-cytidine kinase (UCK) 1 or 2, generating 3'-ethynylcytidine-5'-monophosphate (ECMP). ECMP then undergoes two additional phosphorylations, generating 3'-ethynylcytidine-5'-diphosphate (ECDP) and 3'-ethynylcytidine-5'-triphosphate (ECTP), respectively [1]. ECTP is the final active moiety that inhibits RNA polymerases and exerts the anti-tumor effect (Additional file 1: Figure S1B). Among the three phosphorylation steps, UCKs that mediate the initial phosphorylation are the rate limiting enzymes [2]. In particular, UCK2 is preferentially expressed in cancer cells [3], while UCK1 expression is observed in both cancer and normal cells, explaining the greater anti-tumor effect on cancer cells while sparing normal cells [4–6]. Furthermore, ECyd is a more efficient substrate for UCK2 than for UCK1. In addition, the expression level of not UCK1 but UCK2 is closely correlated with cellular sensitivity to ECyd [6].

Previously, we reported that the combination of ECyd and CDDP showed potent anti-proliferative effects in several *in vitro* cancer cell lines and an *in vivo* xenograft tumor model [7]. Given the remarkable synergistic effect of ECyd and CDDP, we have initiated a Phase I clinical trial combining ECyd and platinum for patients with solid tumors. This novel combination therapy might provide great benefit for patients whose tumor has an intrinsic resistance to CDDP or an acquired resistance after CDDP treatment.

Head and neck (H&N) cancer is the sixth most common cancer worldwide, and around 90% of cases have an epithelial origin that presents as squamous cell carcinoma (SCCHN). Therefore, this histopathological subtype forms the main focus of H&N cancer treatment [8]. CDDP is one of the most effective antitumor agents for the treatment of patients with SCCHN. However, acquired resistance to CDDP is a major obstacle to effective, potentially curative chemotherapy in the clinical management of such patients. Even with new second-line options, including Erbitux, a great need remains for alternatives that can deliver improved survival rates in metastatic disease settings. Effective new agents with different targets and/or mechanisms of action are highly needed as either first- or second-line treatments, in combination with standard chemotherapy or as a monotherapy, especially for metastatic SCCHN [9].

The molecular mechanisms underlying the resistance to CDDP remain unknown in human SCCHN cancers [10]. Several mechanisms found in many drug-resistant cancer cells include a reduction of drug uptake, an increase in drug export, an increase in intracellular detoxification, an increase in DNA repair systems, and so on. With respect to CDDP drug resistance, multidrug resistance-associated protein 2 (MRP2) might be correlated with CDDP resistance [11]. However, in general, multiple reports have shown that CDDP is not a substrate for P-glycoprotein, the product of the multidrug resistance gene MDR, and other members of the ATP-binding cassette superfamily of transporters (ABC transporters). Thus, more detailed studies are required to decipher the mechanism of CDDP drug resistance.

Recently, Vault complex (Vaults) was reported to be associated with CDDP resistance through the elimination of platinum chemotherapeutics from cancer cells [12–16]. Vaults are barrel-shaped cytoplasmic ribonucleoprotein particles composed of multiple copies of three different proteins and a small RNA [17]. The mammalian Vaults are composed of major vault protein (MVP), vault poly ADP-ribose polymerase (VPARP) and telomerase-associated protein 1 (TEP-1), which are complexed with small untranslated vault RNAs (vRNAs) [18–20]. Among the four components, the major component of Vaults is MVP, which constitutes more than 70% of the total mass. Vaults were initially identified as clathrin-coated vesicles, and the first evidence that these structures may contribute to drug resistance was provided when lung resistance-related protein (LRP) was highly expressed in non-P-glycoprotein-mediated drug-resistant cell lines [21]. Subsequent studies showed that LRP is identical to human MVP [22]. Although Vaults are expressed in all human tissues, elevated levels of MVP are found in the gut epithelium, lung epithelium, macrophages, and dendritic cells, which are all typically exposed to xenobiotics [23–26]. These findings imply that Vaults have a role in the defense of such tissues against toxic insults. Consistent with this hypothesis, MVP has been found to be overexpressed in various multidrug-resistant cancer cell lines, together with a range of clinical samples such as H&N, ovarian, lung carcinomas, hepatoblastoma, acute myeloid leukemia, and multiple myeloma [12, 23, 26]. An accumulating number of experimental and clinical investigations have suggested that an elevated expression at the time of diagnosis was an independent prognostic factor for a poor response to chemotherapy and an adverse clinical outcome for a variety of tumor types [16, 27–29]. Because the hollow barrel-shaped structure of the Vaults complex and its subcellular localization have indicated that Vaults are involved in xenobiotic transportation, it was postulated that Vaults contribute to drug resistance by transporting drugs away from their intracellular targets and/or the sequestration of drugs [30, 31]. Although the decisive function of the vRNAs component is not clear, the vRNAs reportedly has the ability to bind chemotherapeutics, suggesting a pivotal role in drug export.

Here, we investigated the antitumor activity of ECyd combined with CDDP in platinum-resistant SCCHN cancer cells named KB/CDDP(T); we found that ECyd suppresses the expression of vRNAs and the CDDP-mediated induction of Vaults, restoring sensitivity to CDDP.

## Methods

### Cells and reagents

KB cells, a human nasopharyngeal carcinoma cell line, and A549 cells, a human lung carcinoma cell line, were obtained from the American Type Culture Collection. CDDP-resistant KB cells, KB/CDDP(T), were established by stepwise dose escalation with CDDP in our laboratory. ECyd was synthesized at Taiho Pharmaceutical Co., Ltd. (Tokyo, Japan). CDDP and CBDCA were obtained from Nippon Kayaku Co., Ltd. (Tokyo, Japan), SN-38 was obtained from Sigma-Aldrich Co., LLC. (Missouri, USA), and ADM was obtained from Kyowa Hakkou Kirin Co., Ltd. (Tokyo, Japan).

### Cell culture and cell survival analysis

KB and KB/CDDP(T) cells were grown in Eagle's Minimum Essential medium containing 10% fetal bovine serum, and A549 cells were grown in F-12 K Medium containing 10% fetal bovine serum. SHIN-3 and HRA cells were grown in RPMI-1640 Medium containing 10% fetal bovine serum. The cells were incubated in a humidified atmosphere of 5% CO_2_ at 37°C. A total of 1×10^3^ cells in 100 μL of culture medium were inoculated into each well of a 96-well plate. After 24 hours of incubation at 37°C, 100 μL of anticancer drugs diluted with the medium to various concentrations were added to each well and the cultures were incubated for 72 hours at 37°C in 5% CO_2_. Cell viability was quantified using a colormetric assay using a Cell Counting Kit-8 (Dojindo, Kumamoto, Japan) [32].

### Drug interaction analysis

A total of 5 x 10^2^ cells in 100 μL of culture medium were inoculated into each well of a 96-well plate. After 24 hours of incubation at 37°C, 50 μL each of ECyd and CDDP diluted with the medium to various concentrations were added to each well; the cultures were then incubated for 24 hours at 37°C in 5% CO_2_, followed by washing each well twice with drug-free medium and 96 hours of incubation with drug-free medium. The cell viability was quantified using a colormetric assay using a Cell Counting Kit-8 (Dojindo) [32]. The presence of an additive or synergistic interaction between CDDP and ECyd was determined using the isobologram analysis reported by Steel and Peckham [33]. The type of interaction between CDDP and ECyd was evaluated by comparing the cytotoxic effects obtained after simultaneous exposures to the drugs with the effects observed after exposure to CDDP or ECyd alone. The interaction indices were calculated using the following equation: interaction index = CDDP c/CDDP e + ECyd c/ECyd e, where CDDP e and ECyd e are the concentrations of CDDP and ECyd that inhibit 50% of the proliferation when used alone, and CDDP c and ECyd c are the concentrations of CDDP and ECyd that produce the same effect when used in combination. According to this method, an interaction index of less than 1.0 indicates a synergistic interaction between two drugs, an interaction index of more than 1.0 indicates antagonism, and an index of 1.0 indicates an additive interaction. The data point in the isobologram corresponds to the actual IC_50_ dose of the combined CDDP and ECyd treatment. If a data point is on or within the three lines, this represents an additive treatment effect, whereas a data point that lies below or above the three lines indicates synergism or antagonism, respectively.

### Preparation of total cell lysates and immunoblot analysis

Whole cell lysates were extracted with the M-PER Mammalian Protein Extract (Pierce, Oregon, USA) containing protease inhibitors. The protein concentrations were determined using a bicinchoninic acid protein assay, and equal amounts of protein were separated using a 7.5% SDS-polyacrylamide gel electrophoresis (SDS-PAGE) and were electroblotted onto polyvinylidene difluoride membranes (Millipore, Massachusetts, USA). After blocking, the membranes were probed with primary antibodies against UCK2, MVP (Novus biologicals, Colorado, USA) and β-actin (abcam, Cambridge, UK). After incubation with horseradish peroxidase-conjugated secondary antibodies, the antigen-antibody complexes were visualized using enhanced chemiluminescence (Pierce). Images were captured using an image analyzer (LAS 3000; Fuji Film, Tokyo, Japan).

### Immunocytochemistry

Cells plated on chamber slides were fixed with ice-cold 100% methanol, quenched with 0.3% H_2_O_2_, and blocked with normal goat serum. After incubation for 30 min with the primary antibodies, anti-MVP, and washing, the biotinylated secondary antibodies were added for 30 min, washed, then followed by preformed avidin DH-biotinylated horseradish peroxidase H complex for 30 min. Slides were then overlaid with DAB, rinsed, dried, mounted, and cover-slipped.

### RNA-mediated interference

Stealth RNA-mediated interference (RNAi; Invitrogen, California, USA) for MVP or stealth RNAi negative control (Invitrogen) was transfected using Lipofectamine RNAiMAX (Invitrogen) according to the manufacturer’s protocol.

### RNA isolation and quantitative real-time reverse-transcription PCR quantification

RNAs were extracted using the RNeasy Mini kit (Qiagen, Venlo, Netherlands). First-strand cDNAs were synthesized using the Quantitect Reverse Transcription kit (Qiagen). Gene expression levels were determined using either the TaqMan Gene Expression Master Mix or the SYBR Green PCR Master Mix on an ABI Prism 7900 platform (Applied Biosystems, California, USA), according to the manufacturer’s protocol. 18S rRNA was used for normalization. The relative quantification of the MVP mRNA and vRNAs was calculated using a comparative cycle threshold method [34].

### *In vivo*study

Tumor fragments approximately 2 mm^3^ in size were transplanted subcutaneously into male BALB/cAJcl-nu nude mice (CLEA Japan, Tokyo, Japan). After reaching a tumor volume of ~150 mm^3^, the mice were randomly assigned to a control group and drug treatment, each consisting of six animals (day 0). CDDP (7 mg/kg) was administered by intravenous injection and ECyd (0.1 mg/kg/hr) was continuously administered using osmotic pumps (Alzet, California, USA) to six mice on day 1. Tumors were excised at 6 hours post-administration. The animal experiments were performed according to the guidelines and with the approval of the Institutional Animal Care and Use Committee of Taiho Pharmaceutical Co., Ltd. The permitted experimental number is 09TC11.

## Results

### Establishment of platinum-resistant KB cells, KB/CDDP(T), through exposure to increasing concentrations of CDDP

**Figure 1:**
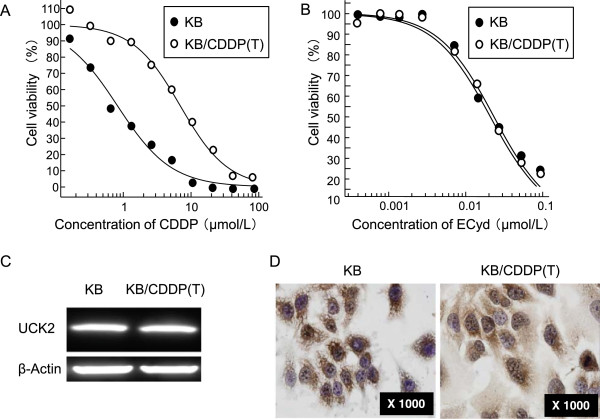
**CDDP resistance shows a similar sensitivity to ECyd. A**, **B)** Sensitivity of KB/CDDP(T) and parental cells to CDDP **(A)** and ECyd **(B)**. Data are shown as the mean (n = 4). **C)** The expression of UCK2 protein in KB/CDDP(T) and parental cells was also analyzed using immunoblot analysis. Equal loading was confirmed by the detection of β-actin. **D)** The expression of UCK2 protein in KB/CDDP(T) and parental cells was analyzed using immunocytochemistry with an anti-UCK2 specific antibody.

KB/CDDP(T) was established as a CDDP-resistant cell line by exposing its parental head and neck cancer KB cells to increasing concentrations of CDDP. We examined the sensitivities to several antitumor agents in both KB/CDDP(T) and parental KB cells. A cytotoxicity and cell viability assay showed a prominent resistance to CDDP in KB/CDDP(T) cells, compared with its parental cells (Figure 1A). The IC_50_ values for CDDP in KB and KB/CDDP(T) cells were 0.82 and 6.92 μmol/L, respectively, meaning that the KB/CDDP(T) cells were more than 8-fold resistant to CDDP than the parental cells (Table 1). Before examining the sensitizing effect of ECyd on the CDDP anti-tumor effect in the resistant cells, we confirmed that the KB and KB/CDDP(T) cells exhibited similar sensitivities to ECyd alone (Figure 1B). We also confirmed that the protein expression of UCK2, which is the rate-limiting enzyme required for ECyd activation to exert its anti-tumor effect, was not changed in KB/CDDP(T) when analyzed using immunoblot analysis (Figure 1C). Immunocytochemistry (ICC) data also indicated no differences in expression or subcellular localization between the two cell lines (Figure 1D). We also assessed the sensitivity to other anticancer drugs (carboplatin [CBDCA], Adriamycin [ADM], and SN-38) between the parental and CDDP-resistant cells. The IC_50_ values of both cells to the anticancer drugs are shown in Table 1. The KB/CDDP(T) cells exhibited resistance not only to CDDP, but also to CBDCA, ADM, and SN-38 without affecting the sensitivity to ECyd. All these agents are known to be substrates for the Vaults to render resistance to these drugs.

**Table 1:** **Cytotoxicities of several drugs against KB and CDDP-resistant cell line, KB/CDDP(T)**

	ECyd		CDDP		CBDCA		ADM		SN-38	
Cell line	IC_50_	Fold	IC_50_	Fold	IC_50_	Fold	IC_50_	Fold	IC_50_	Fold
	μmol/L		μmol/L		μmol/L		μmol/L		μmol/L	
KB	0.022	1.0	0.82	8.4	28.40	7.5	0.022	3.8	0.002	35
KB/CDDP(T)	0.022		6.92		214.13		0.084		0.070	

Cells were exposed to each drug for 72 hr. Data are shown as the mean (n = 4).

### Expression level of Vaults affects the sensitivity to CDDP

**Figure 2:**
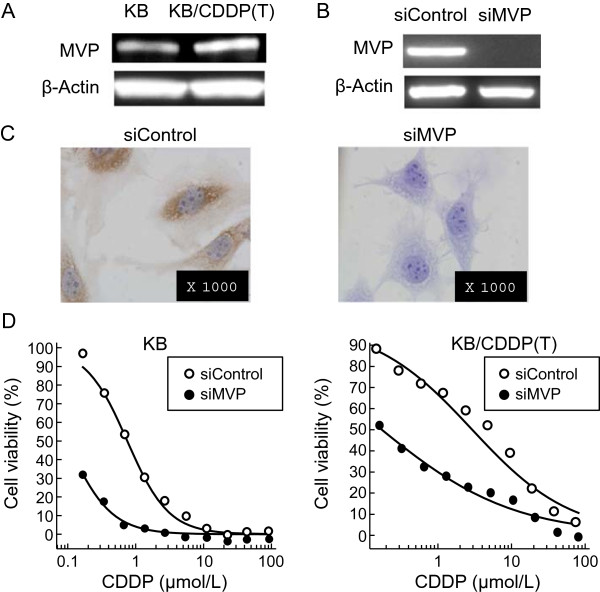
**Silencing of MVP increases the cellular sensitivity to CDDP. A)** The basal expression of MVP protein in KB/CDDP(T) cells and parental cells was analyzed using immunoblot analysis. **B)** The expression of MVP protein in KB/CDDP(T) cells treated with siRNA to MVP or negative control siRNA was analyzed using immunoblot analysis. Equal loading was confirmed by the detection of β-actin. **C)** The expression of MVP protein in KB/CDDP(T) cells treated with siRNA to MVP or negative control siRNA was analyzed using immunocytochemistry. **D)** The sensitivity of KB/CDDP(T) and parental cells treated with siRNA to MVP or negative control siRNA against CDDP was analyzed. Data are shown as the mean (n = 4).

To elucidate the mechanism accounting for the drug-resistance to CDDP, we investigated a ribonucleotide protein, Vaults, since various reports have shown that Vaults expression significantly affects the sensitivity to platinum-based drugs. First, we found that the basal level of MVP was up-regulated in the KB/CDDP(T) cells, compared with the parental cells, when analyzed using immunoblot analysis (Figure 2A). Next, to confirm whether Vaults limited the sensitivity of CDDP in KB/CDDP(T) cells, we assessed the effect of MVP-silencing using RNA interference on the sensitivity to CDDP in KB/CDDP(T) cells. Immunoblot analysis and ICC showed that MVP-silencing sufficiently suppressed the expression of MVP protein in KB/CDDP(T) cells (Figure 2B and C). KB/CDDP(T) cells treated with MVP-siRNA showed a higher sensitivity to CDDP, compared with the cells that were treated with negative control siRNA (Figure 2D). To further confirm this data, we assessed the effect of MVP-silencing in A549 cells, which have a high basal level of MVP expression, and observed a similar sensitization to CDDP in response to MVP-silencing (Additional file 1: Figure S2A and B). In addition, we confirmed that the ERCC1 expression level was not different between KB/CDDP(T) and its parental cells, since multiple studies have shown that ERCC1 induction causes resistance to CDDP (Additional file 1: Figure S3A). These results suggest that the up-regulation of Vaults limit the sensitivity of KB/CDDP(T) cells to CDDP.

### Combination of ECyd and CDDP results in a potent synergistic growth inhibitory effect on KB/CDDP(T)

**Figure 3:**
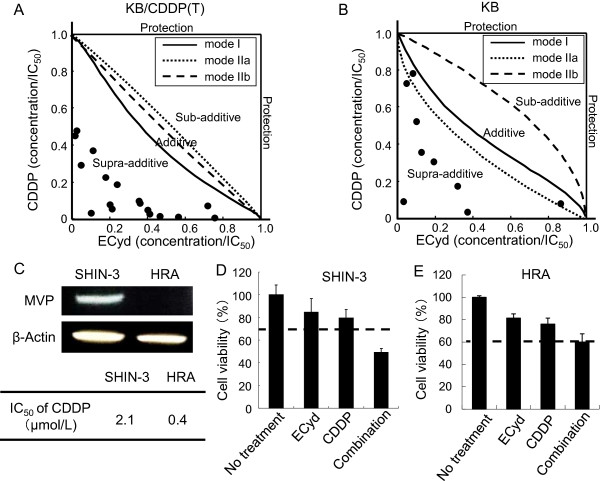
**Simultaneous exposure to ECyd and CDDP causes synergistic cell growth inhibition of cells with higher MVP expression levels.** The effect of 24 hours of simultaneous exposure to ECyd and CDDP was analyzed in parental KB cells **(A)** and KB/CDDP(T) **(B)** cells. The combined effect of ECyd with CDDP was analyzed using an isobologram analysis according to the method described by Steel and Peckham. Data are shown as the mean (n = 4). **C)** The basal expression level of MVP protein in SHIN-3 and HRA cells was analyzed using immunoblot analysis. Equal loading was confirmed by the detection of β-actin. D) The combined effect of 24 hours of simultaneous exposure to ECyd and CDDP was analyzed in SHIN-3 **(D)** and HRA **(E)** cells. The combined effect of ECyd with CDDP was analyzed using bliss independent combination analysis. Data are shown as the mean (n = 4).

Since we previously showed that ECyd inhibits RNA polymerase I-III [1], we hypothesized that ECyd would sensitize the CDDP-resistant cells by inhibiting the CDDP-mediated induction of Vaults expression. To verify this hypothesis, we initially assessed the combined effect of CDDP and ECyd on cell growth. ECyd significantly sensitized the KB/CDDP(T) cells to CDDP in a simultaneous 24 hours combined exposure study. An isobologram analysis (Additional file 1: Figure S4) [33], which can distinguish between the synergistic and additive effects of two compounds, confirmed that the combination of ECyd and CDDP resulted in a remarkable synergistic growth inhibitory effect on KB/CDDP(T) (Figure 3A). In contrast, the combined treatment exhibited an additive or moderate synergistic effect in the parental cells (Figure 3B). These results indicated that ECyd is more efficacious for enhancing the effect of CDDP in CDDP-resistant cells with the induced expression of MVP. In addition, we compared the effect of the combination of CDDP and ECyd between two ovarian cancer cell lines, SHIN-3 and HRA, with and without high MVP expression levels, respectively. When these cells were treated with CDDP alone, the SHIN-3 cells, which have a high MVP expression level, were less sensitive to the drug (Figure 3C). However, in accordance with the data for paired KB cells, the combination of CDDP and ECyd showed a more synergistic effect on the SHIN-3 cells, in which the basal expression level of MVP is higher than that of the HRA cells (Figure 3D and E).

### ECyd decreases vRNAs expression in tumors

**Figure 4:**
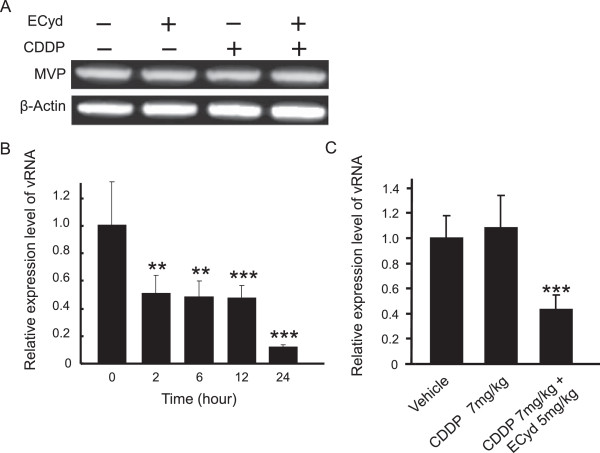
**ECyd decreases the expression of vRNAs, a functionally important component of Vaults. A)** The expression of MVP protein in KB/CDDP(T) cells treated with 7.0 μmol/L (IC_50_ value) of CDDP with or without 0.02 μmol/L ECyd (IC_50_ value) for 24 hours was analyzed using an immunoblot analysis. Equal loading was documented by the detection of β-actin. **B)** vRNAs expression levels in KB/CDDP(T) cells treated with 0.02 μmol/L (IC_50_ value) of ECyd were analyzed using a modified qPCR analysis. The columns are the mean ± SD; **P < 0.01, ***P < 0.001 (n = 3). **C)** vRNAs expression levels in xenograft tumors were analyzed using a modified qPCR analysis. The columns are the mean ± SD; ***P < 0.001 (n = 6).

In order to confirm our hypothesis that ECyd suppresses the expression of Vaults and ECyd up-regulates the cellular sensitivity to CDDP, we assessed the MVP protein expression level after 24 hours exposure of ECyd, CDDP and its combination. However, in contrast to our hypothesis, 24 hours exposure of ECyd, CDDP and its combination had no effect on MVP expression levels (Figure 4A). Next, to investigate whether ECyd inhibits the expression of vRNAs, we analyzed the expression level of vRNAs post treatment with ECyd. Quantification of the vRNAs using RT-PCR, which was specific for the detection of vRNAs [34], revealed that ECyd decreased the expression levels of vRNAs in cultured cells 2–24 hours after ECyd treatment (Figure 4B) *in vitro*. We previously reported that ECyd enhanced the anti-tumor effect of CDDP in a xenograft tumor model *in vivo* [7]. Then, to address whether this hypothesis is active in tumor cells not only *in vitro* but also *in vivo*, we assessed the effect of CDDP and ECyd on the expression levels of vRNAs in nude mice xenograft tumors. Consistent with our *in vitro* data, the co-administration of ECyd statistically decreased the expression levels of vRNAs in nude mice xenograft tumors (Figure 4C), while no induction was observed using CDDP alone.

### ECyd suppresses the induction of MVP protein expression in KB/CDDP(T) cells treated with CDDP

**Figure 5:**
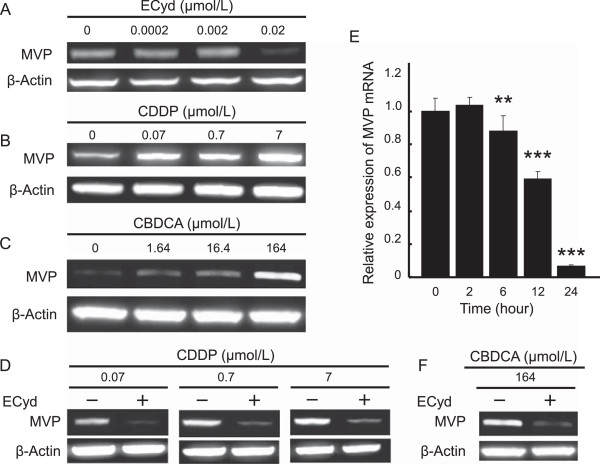
**ECyd cancels the induction of MVP protein expression induced by CDDP treatment. A-C)** The expression of MVP protein in KB/CDDP(T) cells treated with 0–0.02 μmol/L (IC_50_ value) of ECyd **(A)**, 0–7.0 μmol/L (IC_50_ value) of CDDP **(B)** or 0–164 μmol/L (IC_50_ value) of CBDCA **(C)** for 72 hours was analyzed using immunoblot analysis. Equal loading was confirmed by the detection of β-actin. **D)** The expression of MVP protein in KB/CDDP(T) cells treated with 0–7.0 μmol/L (IC_50_ value) of CDDP with or without ECyd (0.02 μmol/L) for 72 hours was analyzed using immunoblot analysis. Equal loading was documented by the detection of β-actin. **E)** KB/CDDP(T) cells were treated with ECyd (0.02 μmol/L) for several terms. The mRNA level of MVP was analyzed using RT-PCR. The Ct value of mRNA was normalized according to that of 18S rRNA as an endogenous control. Columns, mean; bars, SD; **P < 0.01, ***P < 0.001 (n = 3). **F)** The expression of MVP protein in KB/CDDP(T) cells treated with 164 μmol/L (IC_50_ value) of CBDCA with or without ECyd (0.02 μmol/L) for 72 hours was analyzed using immunoblot analysis. Equal loading was documented by the detection of β-actin.

To further examine the involvement of Vaults in the mechanism of CDDP resistance and the restoration of the CDDP effect by ECyd, we assessed the effect of 72 hours exposure to CDDP, ECyd, and their combination on the expression of MVP. We observed that ECyd alone in KB/CDDP(T) decreased the protein expression of MVP (Figure 5A), while CDDP alone significantly increased the protein expression level of MVP in a dose-dependent manner (Figure 5B) [35, 36], although 24 hours exposure of ECyd, CDDP and its combination had no effect on MVP expression levels in KB/CDDP(T) cells (Figure 4A). The exposure to CBDCA for 72 hours also induced MVP protein in KB/CDDP(T) cells (Figure 5C), indicating that MVP expression was generally induced by platinum treatment in the cells. In contrast, ECyd suppressed the CDDP-mediated induction of MVP and reversed the protein expression levels to those similar in the control (Figure 5D) via the inhibition of the mRNA synthesis of MVP (Figure 5E). The CBDCA-mediated induction of MVP expression was also reversed by ECyd treatment (Figure 5F). These results infer that ECyd has a possibility to enhance the anti-tumor effect of CDDP in cells by suppressing the chemotherapeutics-mediated induction of the expression of Vaults, which is the causative molecule for platinum resistance, in addition to Vaults dysfunction by inhibiting vRNAs synthesis.

## Discussion

Although we have previously shown that ECyd enhanced the anti-tumor effect of CDDP [7], the mechanism underlying the sensitization was not clear. This study initially revealed that the enhancement was due to a suppressive effect of ECyd on the Vaults complex that is up-regulated by platinum. We carefully analyzed CDDP-resistant and parental-paired KB cells and identified three supportive observations demonstrating that Vaults is the causative molecule for CDDP resistance in KB/CDDP(T) cells, although several mechanisms of platinum-based drug resistance have been reported [10–16]. First, CDDP treatment induced MVP protein in a dose-dependent manner, which was also observed by CBDCA treatment. Second, MVP-silencing using RNA interference restored the sensitivity to CDDP. Third, the established CDDP-resistant cell line, KB/CDDP(T), expressed a higher MVP expression level at baseline than its parental cell line. Other studies also reported that MVP knock-down and treatment with anti-MVP antibody restored cellular apoptosis in response to CDDP exposure and increased intra-cellular CDDP accumulation [14], supporting our finding that the up-regulation of MVP is the major mechanism of platinum resistance in KB/CDDP(T) cells.

The present study examined the molecular mechanism underlying the sensitizing effect of ECyd in platinum-resistant cells. Although we previously found that ECyd enhances the anti-tumor effect of CDDP in both *in vitro* and *in vivo* models [7], the molecular mechanism explaining this phenomenon remained to be clarified. The strong synergistic effect of the combination of CDDP and ECyd in KB/CDDP(T) cells suggested an antagonistic effect of ECyd on Vaults up-regulation in response to CDDP, resulting in the efflux of CDDP. ECyd seems to exert its suppressive effect on Vaults in two ways, since ECyd is an inhibitor of RNA polymerase I, II, and III [37]. One mechanism is to suppress the expression of vRNAs via the inhibition of RNA polymerase III [38], and the other is to suppress the MVP protein through the inhibition of RNA polymerase II. Especially, the finding that ECyd reduced the expression of vRNAs, followed by the dysfunction of Vaults, in CDDP-resistant cells is critical, since it would allow CDDP to exert an anti-tumor effect restricted by Vaults within 24 hours. Although ECyd alone exhibits an anti-proliferative property in cancer cells, the observation that the 24 hours ECyd/CDDP combination exerts a synergistic effect strongly supports the idea that the distorted function of Vaults contributes to the restoration of sensitivity to CDDP, in contrast to the additive effect of this combination in the parental KB cells. As ECyd significantly sensitized the KB/CDDP(T) cells to CDDP in a simultaneous 24 hours combined exposure study, the molecular mechanisms underlying the ECyd-induced enhancement should exert within 24 hours. Unexpectedly 24 hours exposure of ECyd, CDDP and its combination had no effect on MVP expression levels, however, we found that ECyd drastically decreased the expression of vRNAs, which reportedly have the ability to play a pivotal role in drug export, within 24 hours. Furthermore, the decreased expression levels of vRNAs were also demonstrated in nude mice xenograft tumor without induction of vRNAs in CDDP alone. Therefore, we thought of the Vaults dysfunction by the inhibition of vRNAs expression as the mechanism underlying the ECyd-induced enhancement of CDDP efficacy. In addition to Vaults dysfunction, our additional data also indicated that 72 hours exposure of ECyd decreased the induction of MVP expression. Osmotic stress is known to increase the level of MVP expression [39], and we confirmed that a significant induction of MVP was observed by osmotic stress in KB/CDDP(T) cells (Additional file 1: Figure S5A and B). Similar to the case of the ECyd/CDDP study, ECyd suppressed the up-regulation of MVP protein expression by osmotic stress (Additional file 1: Figure S5C), inferring that the antagonistic effect of ECyd on MVP up-regulation is a general observation, rather than being specific to platinum-mediated up-regulation. Although ECyd is an RNA polymerase inhibitor that is moderately effective even as a single agent in cancer cells, reversing the induction of Vaults, which renders resistance to CDDP, might become the mechanism responsible for the synergistic effect of the combined treatment in addition to Vaults dysfunction by inhibiting the vRNAs synthesis, especially in the long term chemotherapy which reportedly induces the expression of Vaults [12, 23, 26].

Novel therapeutics to overcome CDDP resistance are needed for the treatment of various types of cancer, such as H&N cancer, small cell lung cancer and ovarian cancer [10]. This study implied that ECyd and CDDP could be a reasonable combination therapy for improving the clinical benefit to cancer patients treated with platinum-based therapy. Since we have shown that a synergistic anti-tumor effect is observed in H&N cancer and ovarian cancer cells in the present study, similar to the effect in lung cancer cells that we observed in our previous report [7], it would be interesting to further investigate the effect of this combination in other types of tumors for which the standard medical care is platinum-based therapy. Furthermore, the synergistic effect of ECyd/CDDP is expected to occur preferentially in tumor cells, compared with normal cells. ECyd is activated by UCK2 followed by the inhibition of RNA polymerase I, II and III, which finally leads to the suppression of cancer cell proliferation [6]. Although RNA polymerases are widely expressed in various types of cells, UCK2 is reportedly expressed at a much higher level in tumor cells than in normal cells [6]. This finding suggests that ECyd causes Vaults dysfunction preferentially in tumor cells, minimizing side effects in the normal cells of cancer patients treated with a combination of ECyd and platinum. Clinical trials to determine the maximum tolerated dose of the combination of ECyd and carboplatin was recently completed [40]. Therefore, the clinical outcome of these Phase II trials is eagerly awaited.

In cancer research, the identification of biomarkers to predict the efficacies of therapies has attracted a great deal of attention, given the fact that the clinical benefit of chemotherapeutics is limited in a small portion of patients. We observed that a higher level of MVP expression diminished the anti-tumor effect of CDDP, and the reduction of this effect by ECyd significantly sensitized the resistant cells. In addition to the data indicating that ECyd restores sensitivity to CDDP, a biological mechanism explaining this sensitization has been revealed, in which MVP induction provides resistance to CDDP through the down-regulation of a drug transporter by ECyd. Therefore, the MVP protein level in cancer patients could be explored as a predictive biomarker for identifying patients who may benefit from the combination of ECyd and platinum in future clinical trials.

## Conclusion

**Figure 6:**
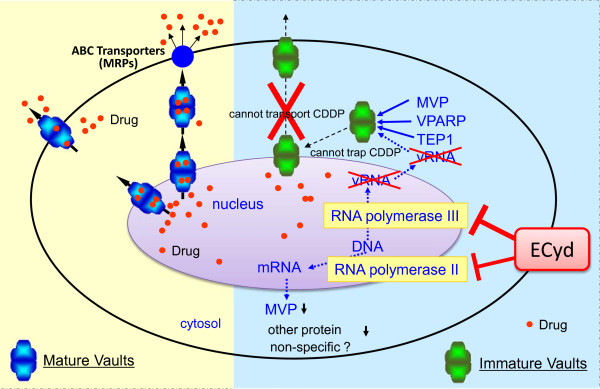
**Possible mechanism for the synergistic combination of ECyd and CDDP through the dysfunction of Vaults.** Vaults seem to be involved in the transport of biomolecules and drugs, and vRNAs, in particular, is thought to be an important component because of its interactions with anticancer drugs. vRNAs is transcribed by RNA polymerase III, which is a target of ECyd, and ECyd gives rise to immature and dysfunctional Vaults that does not contain vRNAs.

We demonstrated the ability of ECyd to cancel the resistance of cancer cells to CDDP by two mechanisms related to the Vaults drug transporter induced by chemotherapeutics, explaining the remarkable synergistic effect of CDDP and ECyd (Figure 6). One is the Vaults dysfunction by inhibiting the vRNAs synthesis as main mechanisms by through of a RNA polymerase III inhibition. Another is the decrease of Vaults expression by through of a RNA polymerase II inhibition. These results suggest that a clinical trial examining the combination of CDDP and ECyd could offer a new strategy for overcoming platinum resistance, which is a problem associated with various types of cancer therapeutics, from both a basic and clinical research perspective.

## Electronic supplementary material

Additional file 1: Figure S1: Structure of ECyd and mechanism by which ECyd inhibits RNA synthesis. **Figure S2.** Silencing of MVP increases the cellular sensitivity of A549 cells to CDDP. A) The sensitivity of A549 cells treated with siRNA to MVP against CDDP. Data are shown as the mean (n = 4). B) The mRNA level of MVP in A549 cells treated with siRNA. **Figure S3.** The Expression levels of ERCC1 and UCK2 are not changed. A) The expression level of ERCC1. The effect of 72 hours exposure of ECyd (B) and CDDP (C) to UCK2 expression. **Figure S4.** Schematic representation of isobologram. The concentration of a 50% cell growth inhibition is expressed as 1.0 on the ordinate and abscissa. The envelope of additivity, surrounded by the mode I , mode IIa, and IIb lines, was constructed from the dose-response curves for CDDP and ECyd. When the data point for a drug combination falls within the envelope of additivity (P2), to the left of the envelope (P1) , to the right of the envelope but within the square or on the square line (P3), or outside of the square (P4), then the combination is respectively regard as additive, supra-additive, sub-additive, or protective. **Figure S5.** ECyd cancels the induction of MVP protein expression induced by treatments in KB/CDDP(T) cells. A and B) The expression of MVP protein in KB/CDDP(T) cells treated with sucrose for 72 hours. C) The expression of MVP protein in KB/CDDP(T) cells treated with sucrose with or without ECyd (0.02 μmol/L) for 72 hours. D) The expression of MVP protein in KB/CDDP(T) cells treated with ADM for 72 hours. (PDF 214 KB)

## Abbreviations

ECyd: 3'-ethynylcytidine
CDDP: Cisplatin
MVP: Major vault protein
UCK: Uridine-cytidinne kinase
H&N: Head and neck cancer
HNSCC: Head and neck squamous cell carcinoma
Vaults: Vault complex
VPARP: vault poly ADP-ribose polymerase
TEP-1: Telomerase-associated protein 1
vRNAs: Vault RNAs
LRP: Lung resistance-related protein
ICC: Immunocytochemistry
CBDCA: Carboplatin
ADM: Adriamycin
MRP2: Multidrug resistance-associated protein 2.
