# Supplementary material for: 3'-Ethynylcytidine, an RNA polymerase inhibitor, combined with cisplatin exhibits a potent synergistic growth-inhibitory effect via Vaults dysfunction
Source: BMC Cancer. 2014 Aug 4;14:562. doi: 10.1186/1471-2407-14-562 (PMC4131025; doi:10.1186/1471-2407-14-562)
Supplement: Supplementary file 1 — Additional file 1: Figure S1: Structure of ECyd and mechanism by which ECyd inhibits RNA synthesis. Figure S2. Silencing of MVP increases the cellular sensitivity of A549 cells to CDDP. A) The sensitivity of A549 cells treated with siRNA to MVP against CDDP. Data are shown as the mean (n = 4). B) The mRNA level of MVP in A549 cells treated with siRNA. Figure S3. The Expression levels of ERCC1 and UCK2 are not changed. A) The expression level of ERCC1. The effect of 72 hours exposure of ECyd (B) and CDDP (C) to UCK2 expression. Figure S4. Schematic representation of isobologram. The concentration of a 50% cell growth inhibition is expressed as 1.0 on the ordinate and abscissa. The envelope of additivity, surrounded by the mode I , mode IIa, and IIb lines, was constructed from the dose-response curves for CDDP and ECyd. When the data point for a drug combination falls within the envelope of additivity (P2), to the left of the envelope (P1) , to the right of the envelope but within the square or on the square line (P3), or outside of the square (P4), then the combination is respectively regard as additive, supra-additive, sub-additive, or protective. Figure S5. ECyd cancels the induction of MVP protein expression induced by treatments in KB/CDDP(T) cells. A and B) The expression of MVP protein in KB/CDDP(T) cells treated with sucrose for 72 hours. C) The expression of MVP protein in KB/CDDP(T) cells treated with sucrose with or without ECyd (0.02 μmol/L) for 72 hours. D) The expression of MVP protein in KB/CDDP(T) cells treated with ADM for 72 hours. (PDF 214 KB) [file 12885_2014_4747_MOESM1_ESM.pdf]

A

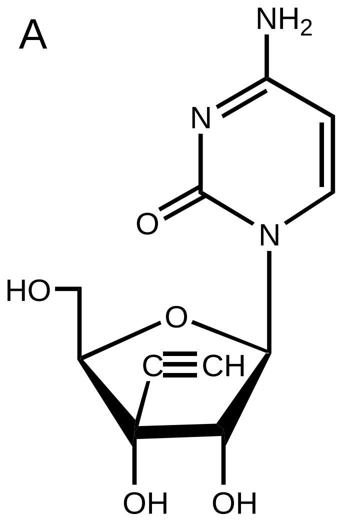


B

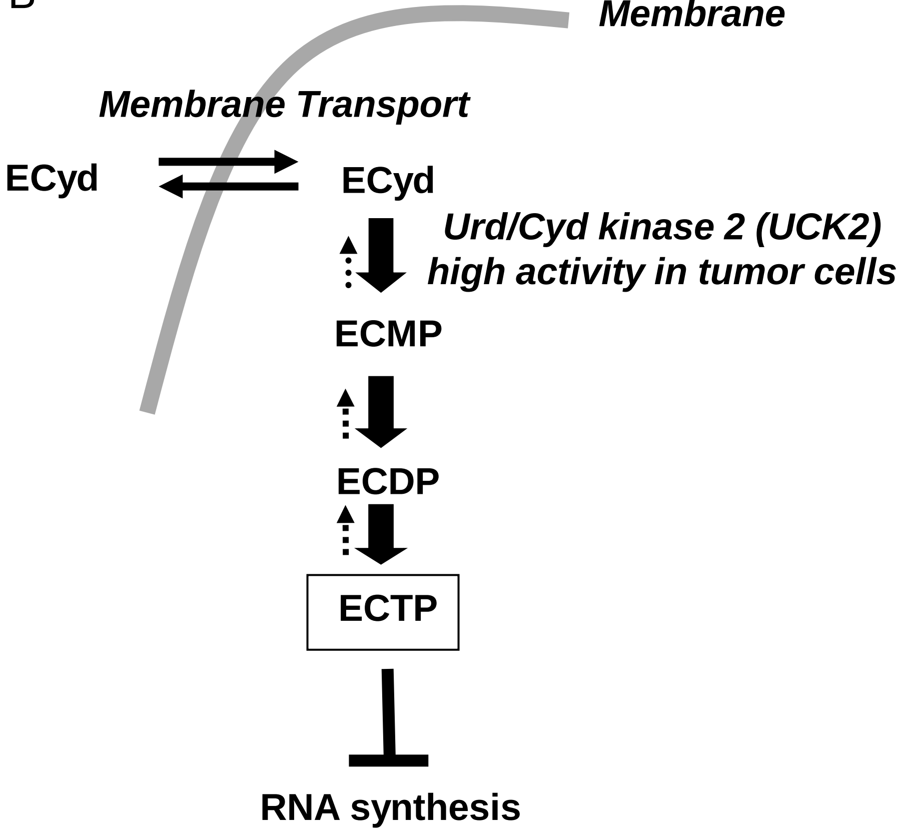


*Supplementary figure S1*

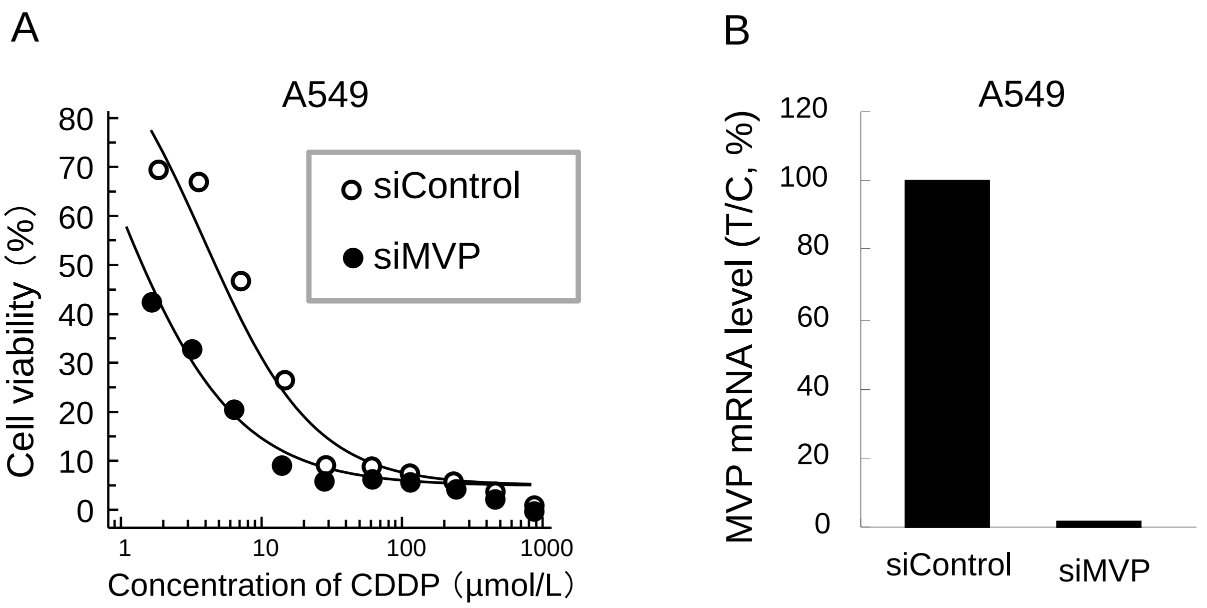


*Supplementary figure S2*

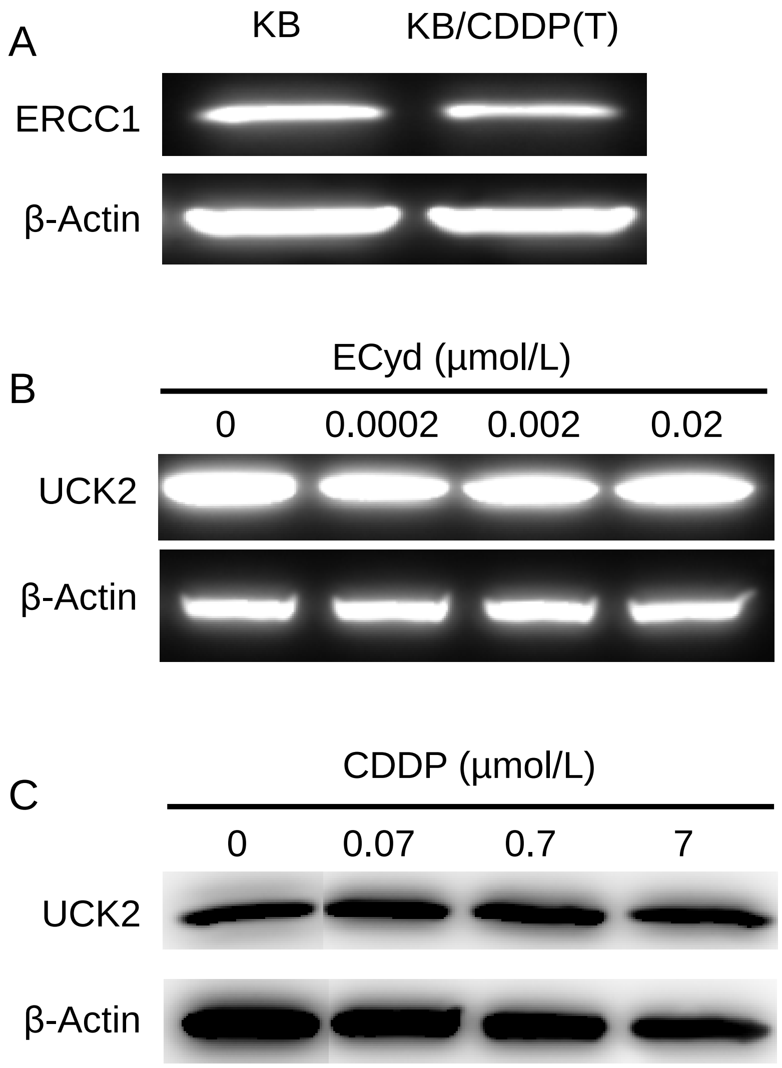


*Supplementary figure S3*

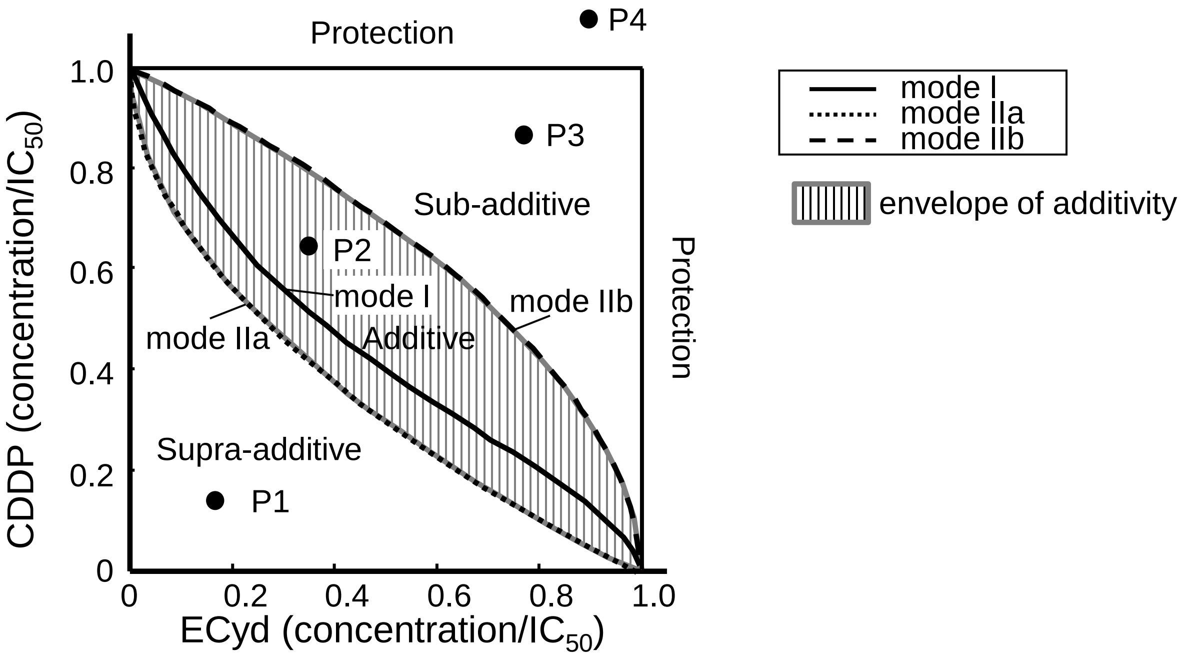


*Supplementary figure S4*

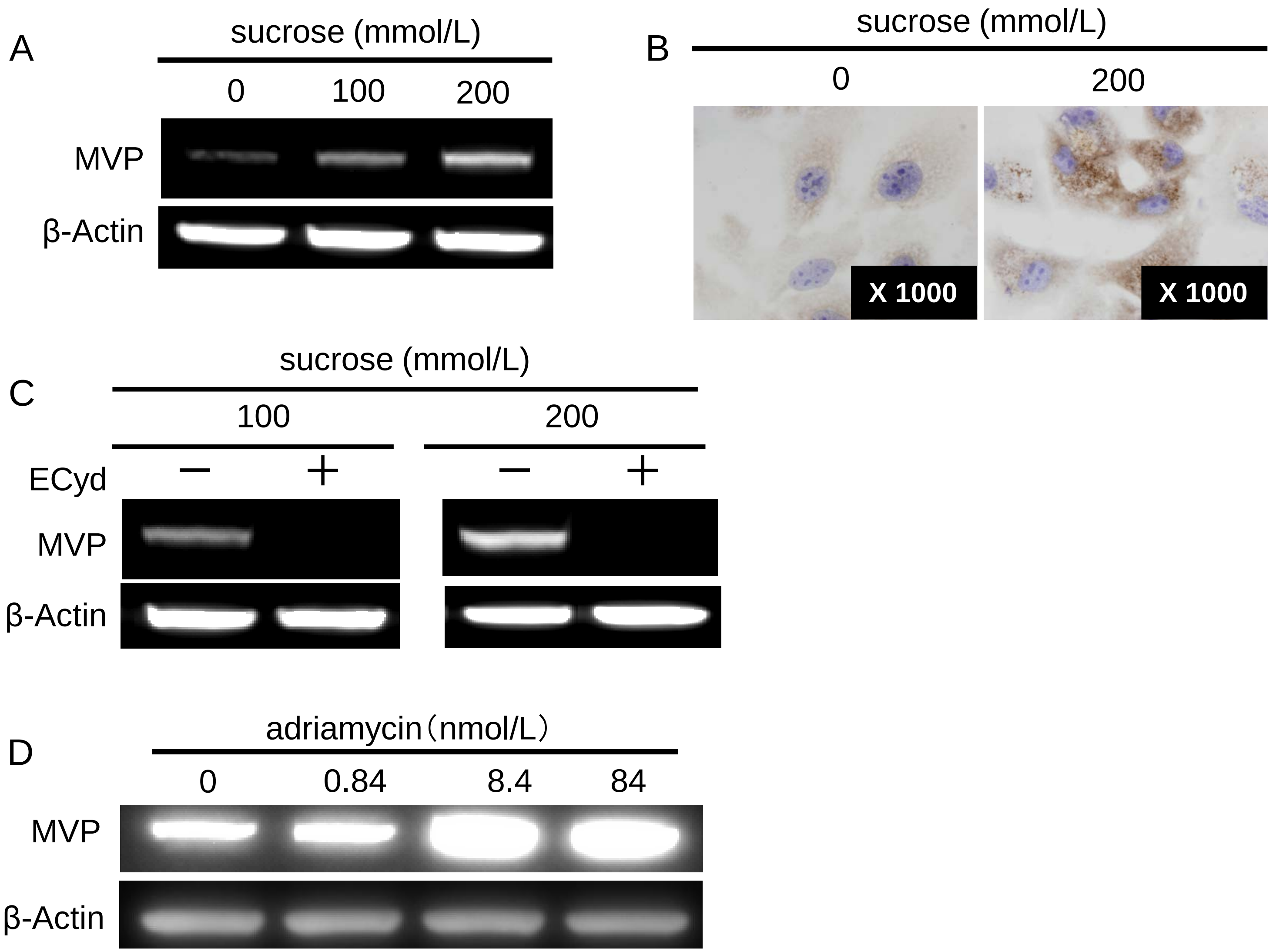


*Supplementary figure S5*
